# The use of dual oxygen concentrator system for mechanical ventilation during COVID-19 pandemic in Sabah, Malaysia

**DOI:** 10.1186/s12245-021-00354-9

**Published:** 2021-05-07

**Authors:** Phee Kheng Cheah, Evelyn Marie Steven, Khai Keam Ng, Muammar Iqbal Hashim, Mohamed Hakimi Abdul Kadir, Nicholas Paul Roder

**Affiliations:** 1 Emergency Retrieval Unit, Emergency and Trauma Department, Sabah Women and Children’s Hospital, Ministry of Health Malaysia, Kota Kinabalu, Malaysia; 2 Emergency Department, Loh Guan Lye Specialists Centre, Penang, Malaysia; 3 Department of Community Emergency Health and Paramedic Practice, Faculty of Medicine, Nursing and Health Sciences, Monash University, Frankston, Victoria Australia; 4 Air Ambulance Victoria, Ambulance Victoria, Village Ave, Traralgon, Victoria Australia

## Abstract

Sabah in Malaysian Borneo is among the Malaysian states which reported a high number of detected COVID-19 cases during the current pandemic. Due to geographical challenges and limited resources, clinicians developed novel strategies for managing patients. The use of a dual oxygen concentrator system for mechanical ventilation is one of the innovations developed by retrieval team members from the Emergency Department (ED) of the Sabah Women and Children’s Hospital. Due to conditions requiring isolation of patients suspected of or positive for COVID-19, high-risk patients were treated in an ED extension area that lacked central wall oxygen. Direct access to oxygen tanks became the only viable option, but ensuring a continuous supply was laborious. The novel setup described within this paper has been used on intubated patients in the ED extension area with moderate to high ventilator settings successfully. This simple setup, designed to meet the limited resources within a pandemic environment, needed only a turbine-driven ventilator, two oxygen concentrators, a 3-way connector, and three oxygen tubing. The application of this setup could potentially save more critically ill patients who are being managed in resource-limited conditions such as in smaller district hospitals or out in the field.

## Introduction

Sabah is a state in Malaysia located on the northern portion of Borneo. A major part of Sabah is surrounded by jungle and mountains which often pose a problem when it comes to transferring and treating patients in rural settings. All hospitals in Sabah started to prepare for the coronavirus disease 2019 (COVID-19) pandemic when the first case was detected on 17 March 2020 in the district of Beaufort. Since then, numbers had increased and hospitals in Sabah began to prepare to treat critically ill patients. Sabah Women and Children’s Hospital (SWACH) started preparation with limited resources, preparing to treat patients in the hospital, while the SWACH retrieval team was placed on standby for the transferring of critically ill patients.

Major hospitals in Sabah have constructed a separate area for treatment and resuscitation of suspected COVID-19 patients using disaster tents and shelters in an effort to segregate high- and low-risk patients arriving at the emergency department (ED). Patients are triaged according to their risk evaluation and if deemed to have a high risk of infection, are directed into the ED extension area [1, 2]. The ED extension was separated into different zones defined as non-critical, semi-critical, and critical. In the extension area, oxygen supply posed a great challenge as there was no central oxygen supply, so teams resorted to oxygen tanks and oxygen concentrators for supply.

For patient transfers, an average built patient with a body weight of 70 kg, with ventilator settings of tidal volume 420 mL, positive end-expiratory pressure (PEEP) 5mmHg, respiratory rate 12/min, and a fraction of inspired oxygen (FiO_2_) 0.5, at least 2 size E oxygen tanks are needed for a 2-h journey. This is equivalent to 16 kg of oxygen [3]. Transfer of patients from district to tertiary hospitals in Sabah is challenging due to the long distances between these hospitals. Our hospital retrieval teams are familiar with transferring ventilated patients using portable battery-operated oxygen concentrators when patients require low ventilator settings due to their ease of use compared to heavy and cumbersome oxygen tanks [4, 5]

Mechanically ventilating patients in everyday situations where there is adequate oxygen supply already requires highly technical skills from trained healthcare workers. Ventilating a patient in the setting of limited oxygen supply and resources would prove an even bigger challenge. At the beginning of the pandemic, we predicted prolonged patient stay in the ED extension area in view of intensive care units (ICU) having to come up with new standard operating procedures (SOP) which involved preparing new isolation bays with delays in receiving patients due to the meticulous steps of donning proper personal protective equipment (PPE). When ICUs are full with critically ill patients, subsequent patients requiring mechanical ventilation become the responsibility of the ED, beyond a duration that would be considered routine. Ventilated patients consume large volumes of oxygen and the changing and replacement of oxygen tanks in the separate treatment area was laborious with the multiple infection control protocols in place. In addition, the supply and refilling of oxygen tanks during the pandemic period were unreliable, and oxygen tanks would often run out during the night. Oxygen concentrators were a great substitute for tank oxygen supply albeit its limited applications as it was not able to supply high-pressure oxygen to our standard oxygen driven ventilators.

In an effort to reduce the use of oxygen tanks in the ED extension area, we paired our turbine-driven ventilator with oxygen concentrators for the ventilation of patients. We limited the single oxygen concentrator technique to patients who needed only low-minute volume settings. However, for patients on high ventilator settings with higher oxygen demand, we used a dual oxygen concentrator system allowing us to double the oxygen inflow to the turbine-driven ventilator. Each oxygen concentrator is only able to contribute a maximum of 5L/min of oxygen flow. Therefore, by connecting two oxygen concentrators using a 3-way connector, we were able to provide a maximum of 10 L/min of oxygen flow to the ventilator.

Our oxygen concentrator, Phillips EverFlo [6], is capable of generating 0.5–5 L/min of continuous flow oxygen. This device produces concentrated oxygen from normal room air for delivery to a patient requiring low-flow oxygen therapy. Oxygen from the air is concentrated using a molecular sieve and a pressure swing adsorption process. It does not have a built-in battery and therefore requires a continuous power supply [7]. The ventilator used was a Weinmann-Meduvent Standard [8], a turbine-driven ventilator capable of receiving low-flow oxygen to a maximum rating of 15 L/min. This ventilator has an oxygen sensor which detects the oxygen concentration being delivered to the patient, and therefore, this reading might vary from breath to breath according to the clinical condition and ventilator settings.

To set the FiO_2_, the minute ventilation administered was used as a guide [9]. According to the desired target oxygen graph provided by the ventilator manufacturer, a 14L/min of oxygen flow will be able to deliver FiO_2_ of 1.0 to the patient at administered minute volume (AMV) 15L/min body temperature and pressure, saturated (BTPS). At oxygen flow of 9L/min, FiO_2_ of 1.0 can be delivered if the AMV was 10L/min BTPS. By combining 2 oxygen concentrators, we estimate that we will be able to deliver 10L/min of oxygen to the ventilator with 10% subtracted for leaking. With this, we estimate that we will be able to safely ventilate a patient using the dual oxygen concentrator system up to a required AMV of 10L/min at FiO_2_ 1.0 [9]. PEEP does not play a role in determining the oxygen concentration delivered [10]. For any patient with a higher ventilatory requirement, this system would not be used. Initial oxygen flow settings are based on the estimation above, but FiO_2_ levels shown on the ventilator will be watched closely and the oxygen flow will be adjusted to achieve the required FiO_2_ once the patient was connected to the ventilator. For clarity, the required FiO_2_ is adjusted by adjusting the flow rate of oxygen on the oxygen concentrator while the delivered FiO_2_ is monitored by looking at the FiO_2_ levels on the ventilator.

**Fig. 1:**
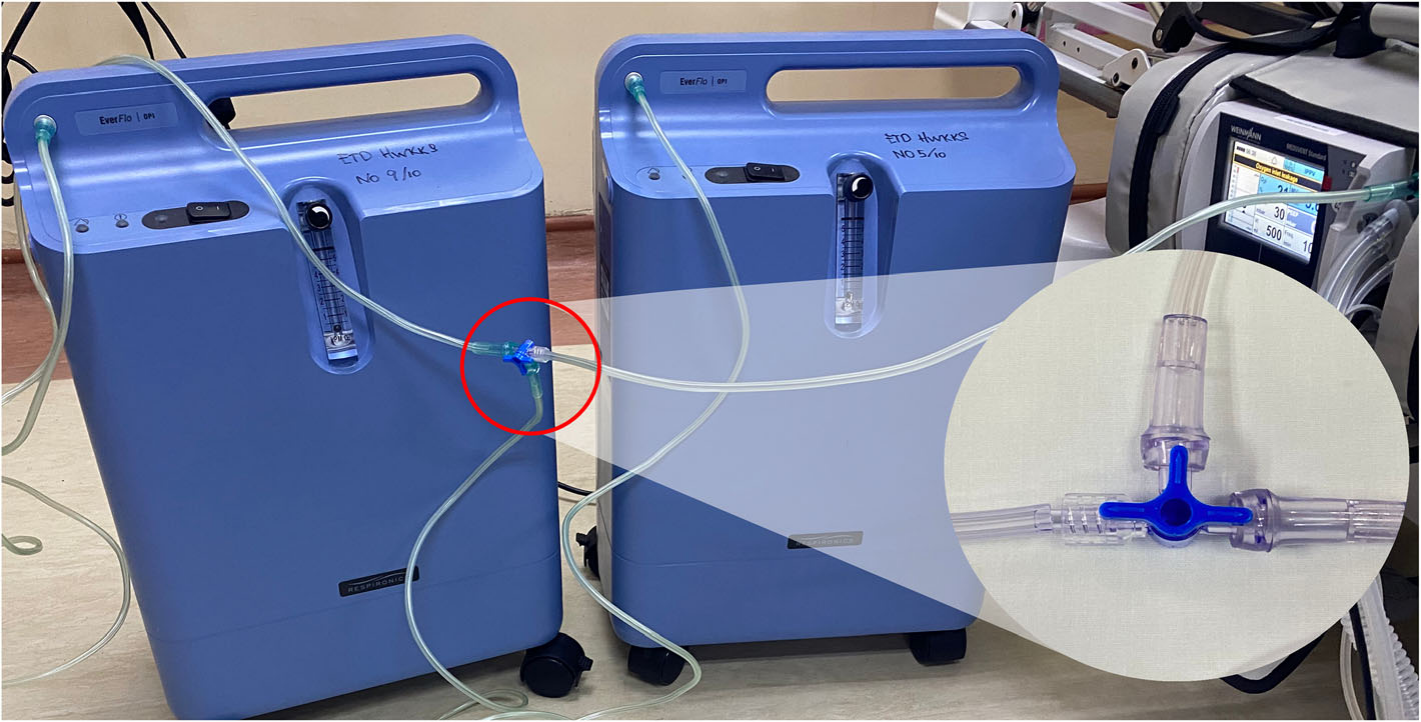
Two oxygen concentrators connected to a low-flow turbine-driven ventilator using a three-way connector

The 3-way connector used to connect oxygen tubing from the two oxygen concentrators was originally designed for the infusion of fluid [11]. While not optimal, its selection was based on limited resources and the lack of a more suitable alternative. The connector was modified to fit the oxygen tubing and act as a connection to direct the flow of both oxygen concentrators into the ventilator. Another two oxygen tubings were connected to the two other ends of the 3-way connector and oxygen concentrators respectively (Fig. 1). The connection system was then checked for leaks, and if present, adjusted or taped. One oxygen tubing [12] is cut at one end in order to attach to the 3-way connector, and subsequently, the other end will be connected to the ventilator.

An oxygen concentrator is a low-maintenance source for supplemental oxygen. In our ED, the oxygen concentrators are able to deliver a maximum of 5L/min of continuous flow oxygen. Continuous flow oxygen is straightforward compared to pulse oxygen. In its conventional usage, continuous flow oxygen concentrators deliver oxygen throughout the breath which leads to a significant fraction of delivered oxygen being lost to the ambient air and never reaching the patient’s lungs. Pulse dose concentrators aim to improve efficiency by limiting oxygen delivery only when patient inspiration can be detected by the trigger mechanism, delivering oxygen as a bolus or pulse early in the breath so that the fraction of oxygen exhaled from the anatomical dead space is reduced. Although usage of pulsed dosed oxygen is advantageous to reduce power consumption, this method of delivery is not suitable for use with this system as the trigger mechanism for pulse oxygen will not be activated when connected to a ventilator.

The main limitation of this particular oxygen concentrator is that a continuous power supply is needed as it does not come with a built-in battery. For transportation of patients, battery-operated oxygen concentrators are used. Although we were still able to achieve the required FiO_2_ with this system, we noticed minimal leaking at the connection between the three-way connector and the oxygen tubing as it was not designed to be used in this manner. To overcome this problem, an oxygen Y-connector [13] could be used for a better seal.

A single oxygen concentrator may be used with a turbine-driven ventilator, but this dual oxygen concentrator system is needed for patients on higher minute volume settings to achieve the desired FiO_2_ of 1.0 in certain patients. This system is suitable for district hospitals, private general practitioner offices, and also on the field to overcome limited oxygen supply. This setup should only be used with turbine ventilators equipped with real-time oxygen sensors due to the possible fluctuation of FiO_2_ with each patient’s breath.

The use of the dual oxygen concentrator system for mechanical ventilation is feasible as a temporary measure until the patient can be transferred to a proper critical care area like the ICU. It is extremely useful in a situation like the COVID-19 pandemic where oxygen supply was limited due to logistics and supply chain constraints. The method described above is a preliminary concept which requires further validation and should be used cautiously if needed.

## Data Availability

Data sharing is not applicable to this article as no datasets were generated or analyzed during the current study.

## Abbreviations

COVID-19: Coronavirus disease 2019
SWACH: Sabah Women and Children’s Hospital
ED: Emergency department
PEEP: Positive end-expiratory pressure
FiO_2_: Fraction of inspired oxygen
ICU: Intensive care units
SOP: Standard operating procedures
PPE: Personal protective equipment
AMV: Administered minute volume
BTPS: Body temperature and pressure, saturated
